# Self-assembling behavior and interface structure in vertically aligned nanocomposite (Pr_0.5_Ba_0.5_MnO_3_)_1-*x*_:(CeO_2_)_*x*_ films on (001) (La,Sr)(Al,Ta)O_3_ substrates

**DOI:** 10.1038/s41598-020-59166-1

**Published:** 2020-02-11

**Authors:** Shao-Dong Cheng, Lu Lu, Sheng Cheng, Lvkang Shen, Ming Liu, Yanzhu Dai, Sheng-Qiang Wu, Shao-Bo Mi

**Affiliations:** 1 State Key Laboratory for Mechanical Behavior of Materials, Xi’an Jiaotong University, Xi’an, 710049 China; 2 School of Microelectronics, Xi’an Jiaotong University, Xi’an, 710049 China

**Keywords:** Surfaces, interfaces and thin films, Structural properties

## Abstract

Heteroepitaxial oxide-based nanocomposite films possessing a variety of functional properties have attracted tremendous research interest. Here, self-assembled vertically aligned nanocomposite (Pr_0.5_Ba_0.5_MnO_3_)_1-*x*_:(CeO_2_)_*x*_ (*x* = 0.2 and 0.5) films have been successfully grown on single-crystalline (001) (La,Sr)(Al,Ta)O_3_ substrates by the pulsed laser deposition technique. Self-assembling behavior of the nanocomposite films and atomic-scale interface structure between Pr_0.5_Ba_0.5_MnO_3_ matrix and CeO_2_ nanopillars have been investigated by advanced electron microscopy techniques. Two different orientation relationships, (001)[100]_Pr0.5Ba0.5MnO3_//(001)[1-10]_CeO2_ and (001)[100]_Pr0.5Ba0.5MnO3_//(110)[1-10]_CeO2_, form between Pr_0.5_Ba_0.5_MnO_3_ and CeO_2_ in the (Pr_0.5_Ba_0.5_MnO_3_)_0.8_:(CeO_2_)_0.2_ film along the film growth direction, which is essentially different from vertically aligned nanocomposite (Pr_0.5_Ba_0.5_MnO_3_)_0.5_:(CeO_2_)_0.5_ films having only (001)[100]_Pr0.5Ba0.5MnO3_//(001)[1-10]_CeO2_ orientation relationship. Both coherent and semi-coherent Pr_0.5_Ba_0.5_MnO_3_/CeO_2_ interface appear in the films. In contrast to semi-coherent interface with regular distribution of interfacial dislocations, interface reconstruction occurs at the coherent Pr_0.5_Ba_0.5_MnO_3_/CeO_2_ interface. Our findings indicate that epitaxial strain imposed by the concentration of CeO_2_ in the nanocomposite films affects the self-assembling behavior of the vertically aligned nanocomposite (Pr_0.5_Ba_0.5_MnO_3_)_1-x_:(CeO_2_)_x_ films.

## Introduction

Complex oxide-based nanocomposite films have attracted considerable research interest due to a variety of functional properties, such as multiferroicity^1^, superconductivity^2–4^, ferromagnetism^5^, and ferroelectricity^6^. The nanocomposite films can be achieved in laminated structure^6–8^, vertical nanocomposite architecture^1–3,5,9^, and three-dimensional framework constructed by interlayering the both^4,10^. Simultaneous phase separation and strain-drived self-assembly processes were first shown to result in vertically aligned nanocomposite (VAN) films related to multiferroic applications and high-temperature superconductor applications^1–4^. Importantly, novel and unprecedented properties could occur in the resultant VAN films, which are not present in the individual phases of the VAN films, e.g., multiferroic in the BaTiO_3_-CoFe_2_O_4_ nanostructures^1^ and enhanced flux-pinning in YBa_2_Cu_3_O_7-δ_ films incorporating self-aligned BaZrO_3_ nanodots and nanorods^2–4^. Moreover, it was found that the self-assembling behavior and the physical properties of the VAN films can be influenced by the phase composition^9,11^ and the film growth parameters (e.g., growth temperature^12,13^, deposition frequency^14^, and substrate termination^15^). In fact, the molar ratio (*x*) of two immiscible phases influences the epitaxial strain of the nanocomposite films on the substrates. For example, the molar ratio of MgO in the nanocomposite (LiFe_5_O_8_)_1-x_:(MgO)_x_ films can tune the structure of LiFe_5_O_8_ nanopillar arrays prepared on fluorophlogopite substrates. As a result, the (LiFe_5_O_8_)_1-x_:(MgO)_x_ VAN films exhibit a higher saturation magnetization (*Ms*), small nonzero coercibity and nonzero remanence compared with the pure LiFe_5_O_8_ film^11^. In addition, the changing of the molar ratio of MgO in the (La_0.7_Ca_0.3_MnO_3_)_1-x_:(MgO)_x_ nanocomposite films on MgO (001) substrates can cause phase transition of La_0.7_Ca_0.3_MnO_3_ from an orthorhombic (0 < x ≤ 0.1) to a rhombohedral structure (0.33 ≤ x ≤ 0.8)^9^.

Additionally, it is believed that two-phase boundaries in the VAN film are of great importance, which provide large vertical interfacial areas and thus induce the coupling effect between the two immiscible phases^16,17^. From this aspect, the VAN films possess superior properties over the single-phase films, e.g., low dielectric loss in VAN BiFeO_3_:Sm_2_O_3_ films^16^. To better understand the performance of the VAN films, it is necessary to explore the self-assembling behavior of the nanocomposite films and interface structure between two immiscible phases at the atomic scale.

The perovskite-type manganites (e.g., Ln_1-*x*_Ba_*x*_MnO_3_ (Ln = La and Pr)) exhibit a wealth of fascinating physical properties and potential practical applications^18,19^. In particular, half-doped Pr_0.5_Ba_0.5_MnO_3_ (PBMO) shows fantastic magnetic behaviors and excellent mixed ionic/electronic conductivity, which enable it potential applications in spintronic devices and solid oxide fuel cell^20,21^. Recently, the (PBMO)_1-x_:(CeO_2_)_x_ nanocomposite films were successfully fabricated, and enhanced magnetic properties (e.g., magnetoresistance and magnetization) were obtained in VAN (PBMO)_1-x_:(CeO_2_)_x_ films compared with the pure PBMO films^22,23^. Furthermore, the microstructure (e.g., lattice mismatch and crystallographic orientation relationship (OR) between PBMO and CeO_2_) of semi-coherent PBMO/CeO_2_ interface in the (PBMO)_0.65_:(CeO_2_)_0.35_ film was presented^23^. Nevertheless, atomic-scale structure and chemical composition of the PBMO/CeO_2_ interface, and strain relaxation behavior of the (PBMO)_1-x_:(CeO_2_)_x_ films on (001)-oriented (La,Sr)(Al,Ta)O_3_ (LSAT) substrates have not been investigated. In addition, the effect of epitaxial strain on the self-assembling growth of the nanocomposite films remains unclear in the (PBMO)_1-x_:(CeO_2_)_x_/LSAT heterosystem.

It is known that the self-assembling growth of VAN films can be accomplished by tuning the epitaxial strain imposed by changing the molar ratio (*x*) of two immiscible phases in the VAN films^9,11^. In the present contribution, to deeply understand the self-assembling behavior and the related structure-property in the (PBMO)_1-*x*_:(CeO_2_)_*x*_ nanocomposite films, the (PBMO)_1-*x*_:(CeO_2_)_*x*_ (*x* = 0.2 and 0.5) films have been prepared on LSAT (001) substrates. We focus our research interest on film-growth behaviors and heterointerface structure investigated by advanced electron microscopy techniques.

## Results and Discussion

**Figure 1:**
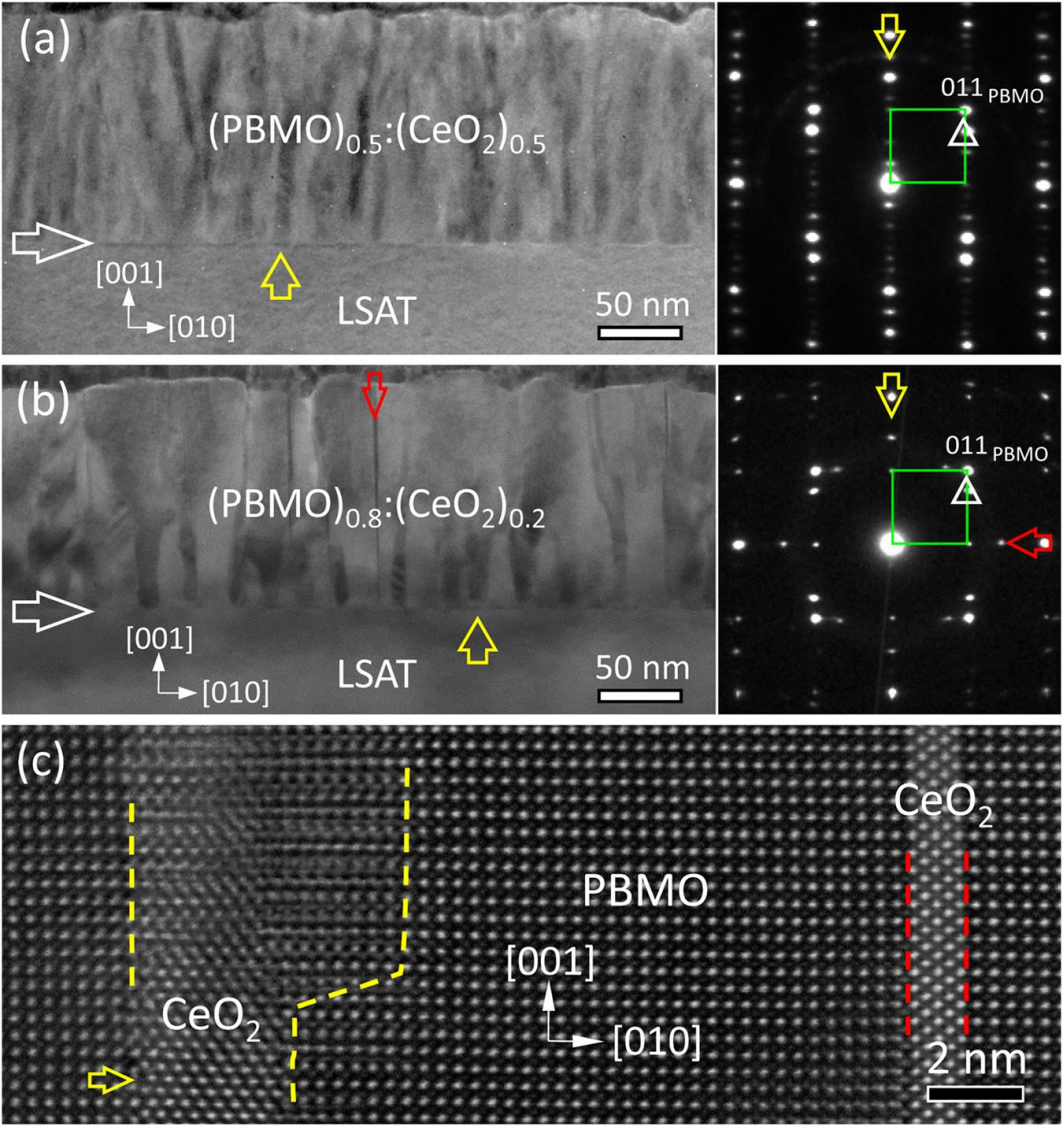
A low magnification BF-TEM image of (PBMO)_0.5_:(CeO_2_)_0.5_ film (**a**) and (PBMO)_0.8_:(CeO_2_)_0.2_ film (**b**) on LSAT substrate, recorded along the [100] LSAT zone axis. The corresponding SAED pattern of the nanocomposite film is inserted. A horizontal white arrow in (**a**,**b**) indicates the film-substrate interface. CeO_2_ nanopillars embedded in PBMO matrix with different dimensions are indicated by a vertical yellow arrow and a vertical red arrow, respectively. In the inserted SAED pattern, the diffraction spots from PBMO matrix are indicated by a green open square. Two sets of diffraction spots from CeO_2_ nanopillars are denoted by a vertical yellow arrow and a horizontal red arrow, respectively. (**c**) A high-resolution HAADF-STEM image of (PBMO)_0.8_:(CeO_2_)_0.2_ film showing two types of ORs between CeO_2_ and PBMO, viewed along the [100] PBMO zone axis. The interfaces is indicated by yellow dashed lines and red dashed lines.

A low-magnification bright-field (BF) TEM image of (PBMO)_0.5_:(CeO_2_)_0.5_ film and (PBMO)_0.8_:(CeO_2_)_0.2_ film on LSAT substrates is displayed in Figs. 1a,b, respectively, viewed along the [100] LSAT zone axis. The thickness of the films is about 130 nm and the film-substrate interface is sharp, as indicated by a horizontal white arrow. No misfit dislocations are observed at the interface of the nanocomposite films on the LSAT substrates. Moreover, CeO_2_ nanopillars embedded in PBMO matrix with a width of about 10–15 nm can be recognized, as indicated by a vertical yellow arrow in Figs. 1a,b. It is noted that narrow CeO_2_ nanopillars with straight sidewalls only exist in the (PBMO)_0.8_:(CeO_2_)_0.2_ film, as demonstrated by a vertical red arrow in Fig. 1b. In most cases, the CeO_2_ nanopillars penetrate the whole film.

In Figs. 1a,b, the inserted SAED pattern taken from the nanocomposite film show the intense and sharp diffraction spots, indicating high-quality epitaxy of the film. The diffraction spots from PBMO matrix can be indexed, as shown by a green open square. The diffraction spots from CeO_2_ nanopillars are visible, as indicated by a vertical yellow arrow. In comparison, one additional set of diffraction spots of CeO_2_ nanopillars appears in the SAED pattern of the (PBMO)_0.8_:(CeO_2_)_0.2_ film, as indicated by a horizontal red arrow in the insert in Fig. 1b. On the basis of the SAED patterns, both (PBMO)_0.5_:(CeO_2_)_0.5_ and (PBMO)_0.8_:(CeO_2_)_0.2_ film have an OR of (001)[100]_PBMO_//(001)[1$\bar{1}$0]_CeO2_ (OR-I) between CeO_2_ and PBMO. Apart from the OR-I, the OR of (001)[100]_PBMO_//(110)[1$\bar{1}$0]_CeO2_ (OR-II) between CeO_2_ and PBMO exists in the (PBMO)_0.8_:(CeO_2_)_0.2_ film. In fact, there is a rotation of 90° between OR-I and OR-II (See Fig. S1 of the Supplemental Material).

Fig. 1c displays a typical high-resolution HAADF-STEM image viewed along the [100] PBMO zone axis, which shows the existence of two types of OR between CeO_2_ and PBMO in the nanocomposite films. It is known that under the HAADF imaging conditions, the atomic columns appear dots in a dark background, and the intensity (*I*) of bright dots is roughly proportional to the square of the atomic number (*Z*) of the atom column^24^. The CeO_2_ nanopillars have a bright contrast in the PBMO matrix. It is found that CeO_2_/PBMO interface can be either semi-coherent or coherent along the film-growth direction, as shown by yellow dashed lines and by red dashed lines, respectively. Interfacial dislocations are visible at the semi-coherent interface, as demonstrated by a horizontal yellow arrow.

**Figure 2:**
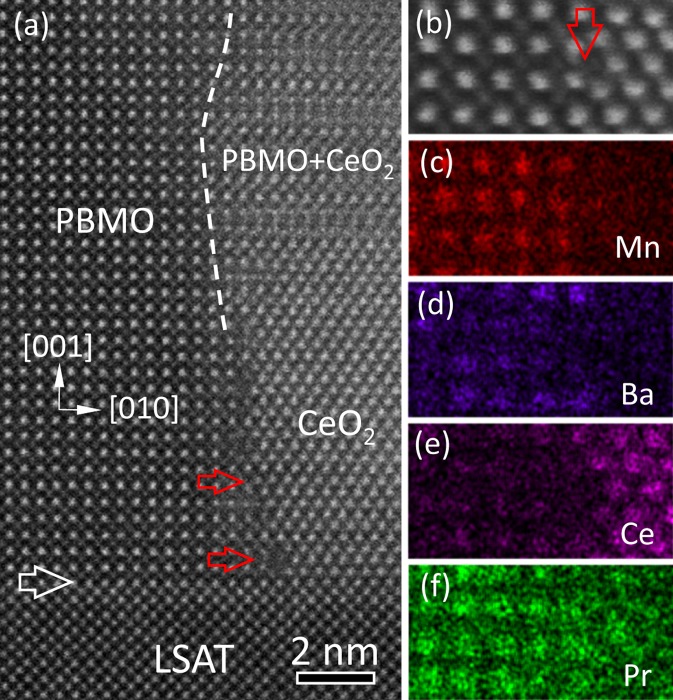
(**a**) An atomic-resolution HAADF-STEM image of the nanocomposite film on LSAT substrate, viewed along [100] LSAT zone axis. A horizontal white arrow indicates the film-substrate interface. A white dashed curved line shows the PBMO/CeO_2_ interface. Interfacial dislocations at the interface are demonstrated by horizontal red arrows. A typical high-resolution HAADF-STEM image of the semi-coherent PBMO/CeO_2_ interface (**b**) and the corresponding EDS maps of Mn-Kα1 (**c**), Ba-Lα1 (**d**), Ce-Lα1 (**e**) and Pr-Lα1 (**f**), respectively. A vertical red arrow in (**b**) denotes the interface.

Fig. 2a shows a high-resolution HAADF-STEM image of the (PBMO)_0.5_:(CeO_2_)_0.5_ nanocomposite film on the LSAT substrate, viewed along the [100] LSAT zone axis. A horizontal white arrow denotes the film-substrate interface. It is found that the coherent growth of PBMO film on LSAT substrate occurs, and the relatively small lattice mismatch (0.7%) between PBMO (*a*_PBMO_ = 0.3895 nm^25^) and LSAT (*a*_LSAT_ = 0.3868 nm^26^) is accommodated by the lattice elastic energy. In addition, the CeO_2_ nanopillar epitaxially grows directly on the LSAT substrate with (001)[1$\bar{1}$0]_CeO2_//(001)[100]_LSAT_, which may be due to the small lattice mismatch (−1.1%) calculated by *Δf* = $\frac{\sqrt{2}{a}_{{\rm{CeO}}2}-2{a}_{{\rm{LSAT}}}}{2{a}_{{\rm{LSAT}}}}$, where *a*_CeO2_ and *a*_LSAT_ are the lattice parameter of CeO_2_ (*a*_CeO2_ = 0.5411 nm^27^) and LSAT, respectively. In contrast, the PBMO/CeO_2_ interface is semi-coherent and interfacial dislocations are observed, as demonstrated by horizontal red arrows.

The atom-scale structure of the semi-coherent PBMO/CeO_2_ interface has been investigated by EDS element mapping^28^. Fig. 2b is a typical high-resolution HAADF-STEM image of the PBMO/CeO_2_ interface. The corresponding EDS maps of Mn, Ba, Ce and Pr are shown in Figs. 2c−f, respectively. In the PBMO matrix, Pr and Ba cations site at the same atomic columns, indicating that A-site disordered PBMO is obtained. According to the EDS measurements, no elemental segregation at the PBMO/CeO_2_ interface. In the CeO_2_ nanopillar, Pr and Ce site at the same atomic columns, implying that Pr^3+^ ions dope into CeO_2_ and partially replace Ce^4+^ ions. The substitution of Pr^3+^ in Ce^4+^ can result in the formation of (Ce,Pr)O_2-*δ*_, and oxygen vacancies generated in the (Ce,Pr)O_2-*δ*_ phase retain the charge balance. The reduced ratio of Pr/Ba in PBMO and oxygen vacancies in the (Ce,Pr)O_2-*δ*_ can influence the transport and magnetic properties of the nanocomposite films^29,30^.

**Figure 3:**
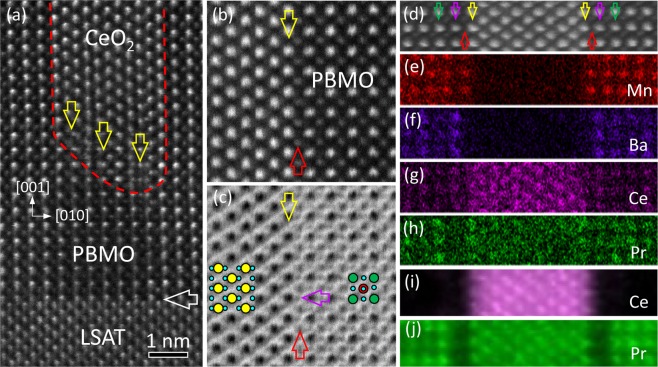
(**a**) A typical HAADF-STEM image of CeO_2_ nanopillar embedded in PBMO matrix with the OR-II on LSAT substrate, viewed along [100] LSAT zone axis. A horizontal white arrow indicates the film-substrate interface. A red dashed curved line denotes the PBMO/CeO_2_ interface. Interfacial dislocations are indicated by vertical yellow arrows. An atomic-resolution HAADF-STEM (**b**) and the corresponding ABF (**c**) image of the coherent PBMO/CeO_2_ interface. Interfacial CeO_2_ and MnO_2_ layer is indicated by a vertical yellow and a vertical red arrow, respectively. The oxygen column in MnO_2_ layer is shown by a horizontal purple arrow in (**c**). A typical HAADF-STEM image of coherent PBMO/CeO_2_ interface (**d**) and the corresponding EDS maps of Mn-Kα1 (**e**), Ba-Lα1 (**f**), Ce-Lα1 (**g**) and Pr-Lα1 (**h**), respectively. (**i**,**j**) The corresponding EELS maps of Ce-M_4,5_ edge and Pr-M_4,5_ edge, respectively. A vertical green, purple, red and yellow arrow indicates the PrO, BaO, MnO_2_ and Ce/Pr atom column at the interface, respectively.

Apart from the OR-I between CeO_2_ and PBMO, CeO_2_ nanopillars with the OR-II in PBMO matrix exist in the (PBMO)_0.8_:(CeO_2_)_0.2_ nanocomposite film. Fig. 3a shows a typical high-resolution HAADF-STEM image of the nanocomposite (PBMO)_0.8_:(CeO_2_)_0.2_ film on the LSAT substrate with the OR-II, viewed along the [100] LSAT zone axis. The PBMO/CeO_2_ interface is denoted by a red curved dashed line. In contrast to the coherent PBMO/CeO_2_ interface along the film-growth direction, the lateral PBMO/CeO_2_ interface is semi-coherent. Interfacial dislocations are observed, as indicated by vertical yellow arrows. It should be noted that CeO_2_ nanopillars do not grow directly on LSAT substrate. A large lattice mismatch between CeO_2_ and LSAT may result in the difficulty in nucleating CeO_2_ nanopillars on LSAT substrate with (110)[1$\bar{1}$0]_CeO2_//(001)[100]_LSAT_.

Atomic-resolution HAADF- and ABF-STEM image of the coherent PBMO/CeO_2_ interface is displayed in Figs. 3b,c, respectively, recorded in the same region simultaneously and viewed along the [100] PBMO zone axis. Under the HAADF- and ABF-STEM imaging conditions, different atomic columns including cations and oxygen can be identified at the interface^24,31^. The interfacial CeO_2_ layer is indicated by a vertical yellow arrow and the terminated layer of the PMBO film is indicated by a vertical red arrow. The contrast of oxygen atoms is visible in the terminated layer in Fig. 3c, as denoted by a horizontal purple arrow. Based on the HAADF- and ABF-STEM observations, the PBMO film terminates at the MnO_2_ layer at the PBMO/CeO_2_ interface.

The structure of the coherent PBMO/CeO_2_ interface has been further examined by atomic-resolved EDS and EELS mapping^32,33^. Figs. 3d−h show a typical coherent PBMO/CeO_2_ interface and the corresponding EDS map of element Mn, Ba, Ce and Pr, respectively. The EDS measurement in Figs. 3g,h indicates that Pr and Ce ions occupy the same site in CeO_2_, which is further confirmed by EELS measurement, as shown in the EELS maps of Ce-M_4,5_ and Pr-M_4,5_ in Figs. 3i,j. At the interface, CeO_2_ atomic plane faces MnO_2_ atomic plane of PBMO. The atomic plane indicated by vertical purple arrows and vertical green arrows in Fig. 3d is BaO and PrO, respectively, which have different intensities from other (Pr_0.5_Ba_0.5_)O planes in PBMO as shown in Figs. 3f,h,j. In other words, interface reconstruction occurs at the PBMO/CeO_2_ interface, resulting in the formation of a single unit-cell thickness of A-site ordered PBMO structure. It is worth mentioning that the distortion of MnO_6_ octahedra is different between A-site ordered and disordered PBMO^34^. In addition, the A-site ordered PBMO occurs a ferromagnetic-paramagnetic transition at about 320 K, while A-site disordered PBMO has *T*_*C*_ ≈ 140 K^25^.

**Figure 4:**
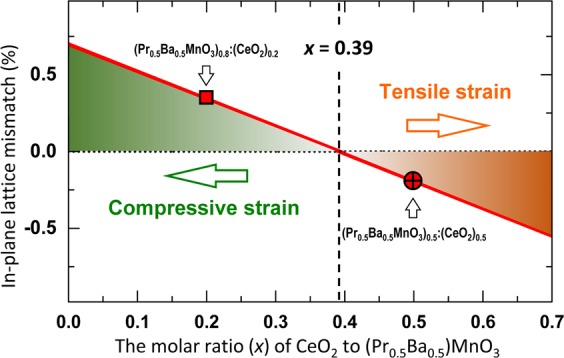
In-plane lattice mismatch between nanocomposite (PBMO)_1-*x*_:(CeO_2_)_*x*_ film and LSAT substrate as a function of the molar ratio (*x*) of CeO_2_ to PBMO in the film for the existence of only OR-I between CeO_2_ and PBMO in the nanocomposite film. The gradual increase of compressive (or tensile) strain is indicated by a horizontal green (or orange) arrow.

It is worth noting that in our work the VAN (PBMO)_1-*x*_:(CeO_2_)_*x*_ (*x* = 0.2 and 0.5) films coherently grow on the LSAT substrates. For the CeO_2_ nanopillars embedded in the PBMO matrix with the OR-I, with the change of the molar ratio (*x*) of CeO_2_ to PBMO, the strain of the VAN (PBMO)_1-*x*_:(CeO_2_)_*x*_ films can be estimated by *Δf = *$\frac{(1-x){a}_{{\rm{PBMO}}}+x\frac{{a}_{{\rm{CeO}}2}}{\sqrt{2}}}{{a}_{{\rm{LSAT}}}}-1$, as shown by a red line in Fig. 4. It can be seen that the strain between the VAN (PBMO)_1-*x*_:(CeO_2_)_*x*_ films and the LAST substrates is close to zero while the molar ratio of CeO_2_ is about 0.39. In addition, the epitaxial strain of VAN (PBMO)_0.5_:(CeO_2_)_0.5_ film and VAN (PBMO)_0.8_:(CeO_2_)_0.2_ has an opposite sign. With the reduction of the molar ratio of CeO_2_ to PBMO, the compressive strain of the nanocomposite films increases, as indicated by a horizontal green arrow.

In the VAN (PBMO)_0.8_:(CeO_2_)_0.2_ film, CeO_2_ nanopillars do appear in the PBMO matrix with either OR-I or OR-II. For CeO_2_ embedded in PBMO matrix with the OR-II, the CeO_2_ nanopillars have few CeO_2_ unit cells in width. Compared with the A-site disordered PBMO, the A-site ordered PBMO at the PBMO/CeO_2_ interface leads to the reduction of lattice parameter (1.8%)^25^, which can partially release the epitaxial strain of the VAN (PBMO)_0.8_:(CeO_2_)_0.2_ film^35^. Additionally, semi-coherent PBMO/CeO_2_ interface with interfacial dislocations appears along the in-plane direction, as demonstrated in Fig. 3a, which can also relax the compressive strain of the VAN (PBMO)_0.8_:(CeO_2_)_0.2_ film on the LSAT substrate. In contrast, the VAN (PBMO)_0.5_:(CeO_2_)_0.5_ film undertakes the tensile strain on the LSAT substrate, as shown in Fig. 4. In the case of the appearance of CeO_2_ nanopillars with the OR-II in PBMO matrix, the tensile strain of the nanocomposite film would be further increased, which is in agreement with the experimental observations that no OR-II occurs between CeO_2_ and PBMO in the VAN (PBMO)_0.5_:(CeO_2_)_0.5_ film.

Compare with A-site disordered PBMO, A-site ordered PBMO has a relative low *Ms* and high magnetoresistance at low temperatures^25^. Nevertheless, considering a very small volume fraction (~20%) of the A-site ordered PBMO in the (PBMO)_0.8_:(CeO_2_)_0.2_ film, the magnetic properties (e.g., *M*_*s*_) of the (PBMO)_1-x_:(CeO_2_)_x_ films on the LSAT substrates would be mainly affected by the epitaxial strain imposed by the CeO_2_ nanopillars within the films^23^. In other words, the volume fraction of CeO_2_ and the crystallographic OR between CeO_2_ and PBMO in the VAN films change the strain state and the magnetic properties of the PBMO film^22,23^. In addition, it was found that the electrical resistivity of the VAN (PBMO)_0.5_:(CeO_2_)_0.5_ film is over 4 times larger than that of the pure PBMO film in our previous work^22^. It is believed that the vertical semi-coherent phase boundary can increase the difficulty of charge carriers transport, and result in an increase of resistivity of the film system^9,36^. The appearance of A-site ordered PBMO at the coherent PBMO/CeO_2_ interface could lead to a decrease of electrical resistivity since A-site ordered PBMO has two orders lower electrical resistivity than A-site disordered PBMO^25^. But, the A-site ordered PBMO in the (PBMO)_0.8_:(CeO_2_)_0.2_ film has a very small volume fraction, which could not strongly affect the resistivity of the VAN (PBMO)_0.8_:(CeO_2_)_0.2_ film^23^. Importantly, our work demonstrates that the epitaxial strain can lead to the formation of A-site ordered PBMO at the heterointerface, which provides a strategy to fabricate A-site ordered PBMO thin films on the substrates (e.g., CeO_2_/YSZ buffered Si substrates^37,38^) by using strain engineering in the heterosystems.

In summary, the VAN (PBMO)_1-x_:(CeO_2_)_x_ films prepared on (001)-oriented LSAT substrates have been systematically studied by advanced electron microscopy. While the VAN film under tensile strain, an OR of (001)[100]_PBMO_//(001)[1$\bar{1}$0]_CeO2_ occurs between CeO_2_ and PBMO in the film. In contrast, the VAN film under compressive strain contains two types of OR, (001)[100]_PBMO_//(001)[1$\bar{1}$0]_CeO2_ and (001)[100]_PBMO_//(110)[1$\bar{1}$0]_CeO2_ between CeO_2_ and PBMO. In addition, interface reconstruction occurs at the coherent PBMO/CeO_2_ interface, resulting in the formation of a single unit-cell-thick layer of A-site ordered PBMO at the interface. Our results demonstrate that self-assembling behavior of the nanocomposite (PBMO)_1-*x*_:(CeO_2_)_*x*_ films can be modulated by epitaxial strain.

## Material and Methods

### Thin film preparation

The composite targets of (PBMO)_1-*x*_:(CeO_2_)_*x*_ (*x* = 0.2 and 0.5) were sintered by a standard ceramic sintering method. The (PBMO)_1-*x*_:(CeO_2_)_*x*_ films were fabricated on (001) LSAT single-crystalline substrates by a KrF (wavelength λ = 248 nm) excimer pulsed laser deposition system with laser energy density of 2.0 J cm^−2^ at 3 Hz. During the film deposition, oxygen pressure is 250 mTorr and substrate temperature is 800 °C.

### Thin film characterization

Cross-sectional transmission and scanning transmission electron microscopy (TEM/STEM) specimens were prepared by focused ion beam (FIB) milling (FEI Helios NanoLab 600i)^39^. Diffraction contrast imaging, selected-area electron diffraction (SAED), high-angle annular dark-field (HAADF) and annular bright-field (ABF) imaging, energy dispersive X-ray spectroscopy (EDS) mapping and electron energy-loss spectroscopy (EELS) mapping were carried out on a probe aberration-corrected JEOL JEM-ARM200F equipped with an Oxford X-Max^N^ 100TLE spectrometer and a Gatan Enfina spectrometer, operated at 200 kV. In STEM mode, a probe size of 0.1 nm at semi-convergence angle of 22 mrad was used. HAADF and ABF detectors covered angular ranges of 90–176 and 11–22 mrad, respectively. EELS collection angle was 72 mrad and energy resolution was 1.2 eV at the dispersion of 0.3 eV/pixel.

## Supplementary information

Supplementary Information.

## Data Availability

All data needed to evaluate the conclusions in the paper are present in the paper and/or the Supplementary Materials. Additional data related to this paper may be requested from the authors.
