# Supplementary material for: Self-assembling behavior and interface structure in vertically aligned nanocomposite (Pr0.5Ba0.5MnO3)1-x:(CeO2)x films on (001) (La,Sr)(Al,Ta)O3 substrates
Source: Sci Rep. 2020 Feb 11;10:2348. doi: 10.1038/s41598-020-59166-1 (PMC7012868; doi:10.1038/s41598-020-59166-1)
Supplement: Supplementary file 1 — Supplementary Information. [file 41598_2020_59166_MOESM1_ESM.docx]

# Supplementary Information

# Self-assembling behavior and interface structure in vertically aligned nanocomposite (Pr_0.5_Ba_0.5_MnO_3_)_1-_*_x_*:(CeO_2_)*_x_* films on (001) (La,Sr)(Al,Ta)O_3_ substrates

Shao-Dong Cheng^1, 2^, Lu Lu^2^, Sheng Cheng^2^, Lv-Kang Shen^2^, Ming Liu^2^, Yan-Zhu Dai^1,2^, Sheng-Qiang Wu^1^, Shao-Bo Mi^1^^[[1]](#footnote-1)^*

*^1^State Key Laboratory for Mechanical Behavior of Materials, Xi’an Jiaotong University, Xi’an 710049, China*

*^2^School of Microelectronics, Xi’an Jiaotong University, Xi’an 710049, China*

Figures S1(a) and (b) display a schematic drawing of EDP of Pr_0.5_Ba_0.5_MnO_3_(PBMO) and CeO_2_, viewed along the [100]_PBMO_ and [1$\bar{1}$0]_CeO2_ zone axis, respectively. Fig. S1(c) shows the composite EDP of (a) and (b), indicating that PBMO and CeO_2_ have the orientation relationship (OR) of (001)[100]_PBMO_//(001)[1$\bar{1}$0]_CeO2_ (OR-I). Fig. S1(d) shows the composite EDP of (a) and (b) with a 90° rotation, indicating that PBMO and CeO_2_ have the OR of (001)[100]_PBMO_//(110)[1$\bar{1}$0]_CeO2_ (OR-II). Fig. S1(e) shows the composite EDP of (c) and (d), indicating the coexistence of two types of OR between PBMO and CeO_2_, (001)[100]_PBMO_//(001)[1$\bar{1}$0]_CeO2_ (OR-I) and (001)[100]_PBMO_//(110)[1$\bar{1}$0]_CeO2_ (OR-II). On the basis of the composite EDPs displayed in (c) and (d), there is a rotation of 90° between OR-I and OR-II. For PBMO and CeO_2_ on the (001)-oriented LSAT substrate, the two types of OR between PBMO and CeO_2_ are schematically illustrated in Fig. S1(f).


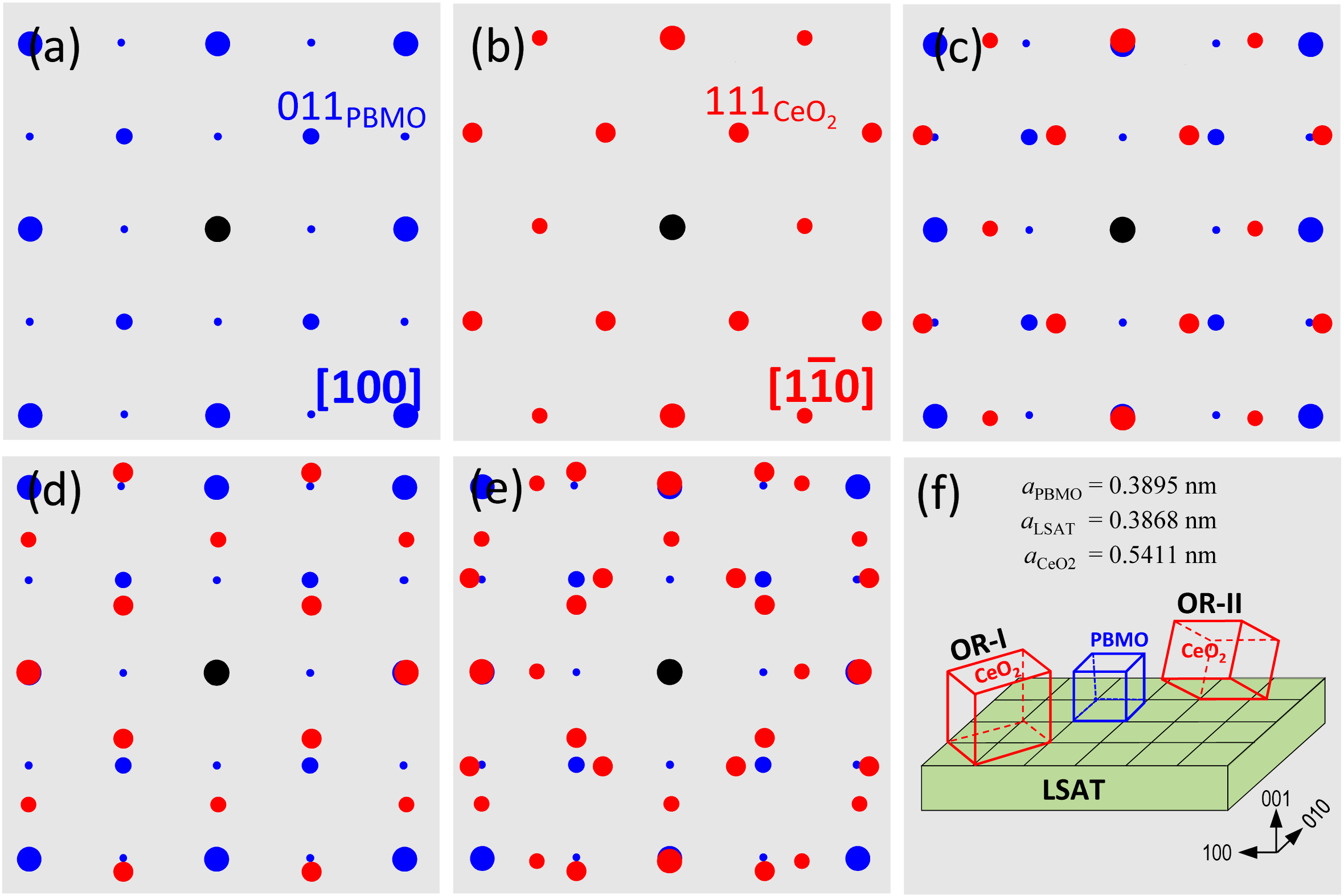


**Figure S1**. (a) and (b) A schematic drawing of EDP of PBMO and CeO_2_, viewed along the [100]_PBMO_ and [1$\bar{1}$0]_CeO2_ zone axis, respectively. (c) The composite EDP of (a) and (b). (d) The composite EDP of (a) and (b) with a 90° rotation. (e) The composite EDP of (c) and (d). (f) A schematic drawing of the OR between PBMO and CeO_2_.

1. * Corresponding author at: State Key Laboratory for Mechanical Behavior of Materials, Xi'an Jiaotong University, Xi'an 710049, PR China.

   Email address: [shaobo.mi@xjtu.edu.cn](mailto:shaobo.mi@xjtu.edu.cn) [↑](#footnote-ref-1)
